# Further Validation of the Crohn’s and Ulcerative Colitis Questionnaire-32 (CUCQ-32) to Measure the Quality of Life in Patients Treated with Biologics Therapy

**DOI:** 10.3390/clinpract12030048

**Published:** 2022-06-10

**Authors:** Laith Alrubaiy, Rafid Sikafi, Hayley Anne Hutchings, Ian Arnott, John Gordon Williams

**Affiliations:** 1 St Mark’s Hospital, Watford Road, Harrow, London HA1 3UJ, UK; rafid.sikafi@nhs.net; 2 Medical School, Swansea University, Swansea SA2 8PP, UK; h.a.hutchings@swansea.ac.uk (H.A.H.); j.g.williams@swansea.ac.uk (J.G.W.); 3 Western General Hospital, Edinburgh EH4 2XU, UK; ian.arnott@nhslothian.scot.nhs.uk

**Keywords:** quality of life, Inflammatory Bowel Disease, Crohn’s disease, ulcerative colitis, Patients Reported Outcomes

## Abstract

Background: Crohn’s and Ulcerative Colitis Questionnaire-32 (CUCQ-32) is a validated questionnaire to measure the quality of life (QoL) in Inflammatory Bowel Disease (IBD). However, it does not have stoma-specific questions and can be lengthy. This study aimed to validate a subset of the CUCQ-32 that would be suitable for patients with a stoma. Methods: Baseline data were collected from a cohort of patients with acute ulcerative colitis who were participating in the CONSTRUCT multicentre clinical trial. A subset of the CUCQ-32 questions was selected by stepwise regression. Further validation was examined using data from the UK IBD biological therapies audit. Construct validity was carried out using the EuroQol 5 dimensions (EQ5D) questionnaire, Simple Clinical Colitis Activity Index (SCCAI), and the Harvey–Bradshaw Index (HBI). Results: Using the data from 124 patients, a short-version questionnaire (CUCQ-12) was developed. Data from 484 patients with IBD (382 patients with Crohn’s disease, 76 patients with ulcerative colitis, and 26 patients with IBD-Unclassified) and 61 patients with stoma provided further validation of the CUCQ-12. A literature review and an expert focus group identified supplementary stoma-specific questions for the CUCQ-12+. The CUCQ-12+ demonstrated excellent internal consistency (Cronbach’s α = 0.86); established effective reproducibility (intra-class correlation coefficient = 0.74); correlated well with the EQ5D (r= −0.48), HBI (r = 0.45), and SCCAI (r = 0.43); and represented good responsiveness statistics (>0.5). Conclusions: CUCQ-12+ is a valid and reliable QoL measure used for all patients with IBD in clinical practice, including patients with a stoma.

## 1. Introduction

Inflammatory Bowel Disease (IBD) is known to impair quality of life (QoL) [1,2,3] and cause a substantial burden to patients, their families and society [4,5,6]. Alternating relapsing and remitting episodes characterise the chronic, incurable nature of the condition with a wide variation in symptom severity and complexity.

QoL questionnaires are important outcome measures that help obtain insight into patients’ perceptions of their condition and how their care may influence this [7]. The tools used to assess QoL are generally disease-specific or generic measures. Disease-specific measures appraise domains unique to a given disease and are therefore regarded as more sensitive to changes in the patient’s state of health [8].

Several condition-specific QoL measures for patients with IBD [9,10,11,12,13] have been developed in the past few decades. Despite the availability of these measures, they are not used in conventional daily clinical practice and are, generally, neglected in large IBD registries due to the limitations on the extent of data collection and the high volume of IBD patients looked after in outpatient clinics. Simple-to-use, quick-to-complete, and concise tools could reinforce the routine use of QoL measures in patient care.

In severe cases of IBD, patients may need hospitalisation and, on occasion, surgery resulting in a stoma, which may have a negative impact on QoL [14,15,16,17,18]. Although generic QoL measures have been used, they are not specific for patients with a stoma [14,19,20]. Aspects of QoL that are important to patients with a stoma, such as social discomfort and smell, simply have not been included in the current QoL measures [21]. With the emerging new therapies and surgical techniques, a QoL measure in IBD that includes stoma-specific questions is necessary to compare various stoma types, treatments, and interventions in IBD. Studies on patients with a stoma have used generic QoL questionnaires or cancer-related QoL questionnaires [21,22,23,24,25]. It is essential to mention that none of the currently available QoL measures in IBD has specifically included stoma-related items. 

The Crohn’s and Ulcerative Colitis Quality of Life Questionnaire-32 (CUCQ-32) is a validated tool to assess the QoL of patients with IBD [26]. However, it contains 32 questions, which some clinicians may perceive to be lengthy for use in routine clinical practice. 

This study aimed to derive and validate a shorter version of the CUCQ-32 that encompasses the different presentations and severity of IBD, including patients with a stoma. The questionnaire will have as few items as possible to be completed while maintaining its effectiveness through proven validity and reliability on rigorous psychometric testing [27] using data from the UK IBD biological therapies audit. 

## 2. Materials and Methods

### 2.1. Developing a Shorter Version of the CUCQ

The first phase of this study was to derive a shorter version of the CUCQ-32. Baseline data were collected from a cohort of patients with acute ulcerative colitis who were participating in the CONSTRUCT (Infliximab versus ciclosporin for steroid-resistant acute severe ulcerative colitis) multicentre clinical trial [28]. Consented patients from 22 hospitals across the UK were asked to complete the Crohn’s and Ulcerative Colitis Quality of Life Questionnaire-32 (CUCQ-32). 

Stepwise regression was used to identify the best combination and the minimum number of CUCQ-32 questions required to accurately reflect the total CUCQ-32 score [29,30].

A literature search was carried out to identify the changes and supplementary questions needed to cover patients with a stoma. An expert focus group of two gastroenterologists, two colorectal surgeons, and two outcome measurement experts guided the process.

Following the reduction of the CUCQ by stepwise regression, we undertook cognitive interviews and piloted the short version of CUCQ-32 on a group of 20 patients (10 patients with Crohn’s disease (CD) and 10 with ulcerative colitis (UC)) with the stable disease to ensure stable disease that the shortened questionnaire was clear to patients. A semi-structured approach using “think aloud” technique was followed to encourage patients to participate and identify items to be included in the stoma-specific questionnaire. The patients were shown the questionnaires at each session and asked to contribute ideas and preferences about the questions’ format, layout and response categories. This was done to ensure the questions addressed all IBD-related issues that are considered necessary by patients and provide feedback regarding the content of the shortened CUCQ and its stoma-specific supplementary questions. 

### 2.2. Validation of the Short Version of CUCQ

We used the short version of CUCQ to collect data from patients who received biological therapy for their IBD treatment as part of the UK IBD biological therapies audit. All hospitals from across the UK were invited to participate. The study included patients with diagnosed IBD, ulcerative colitis (UC), Crohn’s disease (CD), and IBD-type unspecified (IBD-U). Patients of all ages were included. 

Patients were asked to complete the short CUCQ questionnaire and the EuroQol 5 Dimensions (EQ5D) generic QoL measure [20]. The treating IBD specialists were asked to fill in a simultaneous assessment of the patient’s current disease activity, using the Harvey–Bradshaw Index (HBI) for CD [31] and Simple Clinical Colitis Activity Index (SCCAI) for UC [32]. Each patient was asked to rate their bowel condition on a scale of 0 to 100 (0 being the worst possible and 100 being the best possible bowel condition). A sub-sample of patients who returned to the clinic for a follow-up appointment was asked to complete the same questionnaires a second time. 

### 2.3. Psychometric Analysis

We applied the Statistical Package for Social Sciences (SPSS) version 22 to perform the necessary psychometric testing:

Stepwise regression: We used stepwise regression to derive the shorter version of CUCQ by identifying the combination of questions that best predicted the total score of CUCQ [29,30].

Assessing internal consistency: Cronbach’s α was used to evaluate the internal consistency of the short CUCQ. In order to achieve a satisfactory consistency, Cronbach’s α must surpass 0.7 [33]. We eliminated specific questions if their correlation to the total score (item–total correlation) was below 0.2 [27,33] or over 80% of responses were similar (maximum response ratio), due to the low sensitivity of these questions in differentiating between the severity of symptoms [27,33].

Assessing validity: We used one generic QoL measure EuroQol 5 dimensions (which has two components: the 5-item descriptive (EQ5D) and a visual analogue scale (VAS)) as well as two IBD specific tools (SCCAI and HBI) to evaluate the effectiveness of the short CUCQ. To ensure the short CUCQ is an accurate measure of the impact of IBD on a patient’s health, it would need to show a positive correlation (r > 0.4) with QoL measures and disease severity [33,34].

Assessing reproducibility (intra-class correlation): We assessed the reproducibility of the short CUCQ by analysis of a retest questionnaire to a subgroup of patients who came for a follow-up visit after the first completed the short CUCQ questionnaire. In the follow-up questionnaires, we asked patients an anchor question asking about their IBD condition and symptoms on a 100-point scale. To check consistency between the two sets of responses, only patients who reported no change in their bowel condition on the 100-point scale (a change less than 10 points) compared to their first visit were included in the reproducibility analysis. We applied the intra-class correlation coefficient to evaluate the reproducibility of scores for these stable patients [35]. An intra-class correlation between the two sets of questionnaires should surpass 0.70 to reach adequate reproducibility [36].

Assessing responsiveness: We analysed responsiveness in retested patients that revealed a change in their bowel condition on the 100-point scale. We calculated three common responsiveness statistics: effect size, standardised response mean (SRM), and the responsiveness ratio (RR) [35]. The acceptable value for responsiveness ratio is 0.5 or 50% [27,28,29]. The responsiveness ratio (RR) is the ratio of the mean change in score of patients with improvement or deterioration at the baseline and second assessments to the standard deviation (SD) of scores of the stable group at baseline [35,37,38]. Effect size (ES) is calculated as the mean change in score of patients with improvement or deterioration at the baseline and second assessments divided by the standard deviation of scores at baseline (of the group that had a change in the health status). The standardised response mean (SRM) is calculated by dividing the mean change in scores of patients with improvement or deterioration at the baseline and second assessments by the standard deviation of the differences in scores between the two visits [25]. The SRM differs from ES because the denominator is the SD of change in scores (rather than baseline scores). The effect size value of 0.2 is considered small, 0.5 is medium, and ≥0.8 is large [27].

Defining the minimally important change (MIC): We used the difference in patients’ rating of their bowel condition change in the second visit as an anchor to calculate the MIC. The MIC was the mean change of the scores of patients who reported a difference of less than 10% in their bowel condition on the 100-point scale. 

## 3. Results

### 3.1. Development of the Short CUCQ

We carried out stepwise regression using the data from 124 patients with ulcerative colitis (UC) who participated in the CONSTRUCT multicentre clinical trial [17] between May and December 2010. Stepwise regression of the 32 questions of CUCQ-32 identified 12 questions that together accounted for 95% of the variance in the total CUCQ-32 scores between patients. The shorter version of CUCQ-32 was called the Crohn’s and Ulcerative Colitis Quality of life questionnaire-12 (CUCQ-12) (Table 1 and Appendix A). The pilot study on 10 patients with stable IBD aged 30–55 y demonstrated clarity of the questions to patients.

The CUCQ-12 questions addressed the following 12 dimensions: sleep, appetite, energy level, urgency, bloatedness, incomplete emptying of bowels, blood in stool, generally unwell, faecal incontinence, nocturnal diarrhoea, passing flatus, and abdominal pain. The total CUCQ-12 score ranged from 0 (best) to 168 (poor), with each question scored between 0 (best) and 14 (poor). These numbers correspond to the number of days affected by a parameter in a fortnight. 

To include patients with a stoma, the literature review and the expert focus group identified 10 supplementary questions that were added to the CUCQ-12. These questions (Table 1) covered body and sexual image, stoma function, and skin irritation. The CUCQ-12 questionnaire was called the Crohn’s and Ulcerative Colitis Quality of Life Questionnaire-12 plus (CUCQ-12+).

No additional questions were recommended as a result of the pilot study. The mean completion time for CUCQ-12 was 5 min (±3 min).

### 3.2. Validation of the CUCQ-12+

A total of 507 patients from 181 hospital sites completed the CUCQ-12 questionnaires as part of the UK IBD biological therapies audit between September 2011 and February 2014. We set a cut-off of 9 questions (75%) for the inclusion of individual responses. The data from 23 patients who answered less than 9 questions were excluded from the study. Missing data were replaced by means of the remaining questions scores for each patient. The data from the remaining 484 patients (382 CD, 76 UC, and 26 IBD-U) were analysed. None of the questions were mainly not answered. The spectrum of disease severity and the characteristics of the patients’ sample at baseline are summarised in Table 2 and Table 3.

The homogeneity of CUCQ-12 was excellent, with Cronbach’s α equal to 0.864. The correlations of each of the 12 items with the total score exceeded 0.2 (Table 4). Reassuringly, none of these correlations was greater than 0.8, showing that all items added extra information. In addition, no single response was chosen by more than 80% of patients, showing that all items achieved some discrimination. Using the 15% recommended value for the percentage of patients who scored the highest or the lowest scores [26], there were no ceiling or flooring effects in the CUCQ-12.

CUCQ-12 had significant (*p* < 0.05) and demonstrated good correlations with generic QoL measures EQ5D (r = −0.48) and VAS (−0.39). Correlation was also good at disease-specific levels (r = 0.51 in CD, r = 0.56 in UC) but was slightly low in IBD-U (r = 0.303) with *p* = 0.151. Correlation with disease severity indices were also good with HBI (r = 0.45) and SCCAI (r = 0.43) (Table 5).

A total of 61 patients (60 with CD and 1 with UC) had a stoma and completed the CUCQ-12+. The internal consistency of the supplementary stoma questions was excellent (Cronbach’s α = 0.84). None of the items correlated more than 0.8 or a single response of more than 80%. The supplementary stoma questions moderately correlated with the rest of the CUCQ-12 questions (r = 0.59, *p* < 0.05). However, we noted a positive but poor correlation of the stoma questions with the generic QoL measures, with EQ5D (r = 0.03) and VAS (r = 0.19).

Using stepwise regression analysis, we shortened the 10 supplementary stoma questions to 5 questions that represent more than 95% of the 10 questions variance (Table 1). The final version of the CUCQ-12+ included 12 questions related to IBD and 5 questions that are stoma-specific (Appendix A).

A subgroup of 36 patients repeated and returned the CUCQ-12 in their second visit within 1 y (the average return time was 3 m). In the reproducibility analysis, we included 11 patients who reported no change in their bowel condition, demonstrating a good intra-class correlation coefficient of 0.75. The remaining 25 patients who reported changing bowel conditions were included in the responsiveness analysis. The responsiveness statistics for CUCQ-12 were good (RR = 0.52; SRM = 0.51; and the effect size (ES) = 0.57). Using the patients’ change in bowel condition as an anchor, we defined the minimally important change (MIC) of CUCQ-12 as a change of 9 points.

## 4. Discussion

Evaluating QoL in patients with IBD is essential in order to assess patient response to treatment. In the last few decades, there has been a rapid increase in the number of measures to evaluate the QoL in patients with IBD [9,10,11,12,13]. However, these measures were limited to clinical trials and are not being used in routine clinical practice. This is primarily because the questionnaires are lengthy and there are often licensing costs associated with using some of the questionnaires.

The CUCQ-32 is a validated measure for use in mild and moderate IBD [26]. CUCQ-32 was a valid and reliable measure for QoL in IBD. However, it contains 32 questions, and its length may affect its routine use in clinical practice. CUCQ-32 was not validated in patients with a stoma. Therefore, this study aimed to determine the feasibility of developing and validating a shorter version of the CUCQ for use in patients with different presentations of IBD, including patients with a stoma.

The first stage of the study involved identifying the questions that could be included in a shortened version of the CUCQ. We used data from a cohort of patients with acute UC who participated in the CONSTRUCT clinical trial. The resulting 12-item questionnaire, the CUCQ-12, predicted 95% of the total CUCQ-32 score. The literature review and the expert focus group identified supplementary questions that included patients with a stoma, which covered body and sexual image, stoma function, and skin irritation. The CUCQ-12 questionnaire was called the Crohn’s and Ulcerative Colitis Quality of Life Questionnaire-12 plus (CUCQ-12+).

In stage two of our study, we assessed the data from the 484 respondents of the CUCQ-12 questionnaire that were recruited as part of the UK IBD biological therapies audit [39]. The results demonstrated that CUCQ-12 had good reliability and validity. It displayed excellent internal consistency with a Cronbach’s α value of 0.84, above the 0.70 thresholds proposed by Nunnally [34]. The CUCQ-12 showed good correlations with other QoL measures and disease-specific severity indices. An intra-class correlation coefficient of 0.75 demonstrated satisfactory reproducibility. The good responsiveness ratio, SMR and ES of more than 0.5 suggests that the CUCQ-12 is responsive to changes in patients’ clinical conditions and suitable for longitudinal monitoring of QoL. A change of 9 points in the total CUCQ-12 score was found to be the minimally important change as perceived by patients.

A total of 61 patients had a stoma and completed the CUCQ-12+ questionnaire. The results showed that the supplementary stoma questions had a good internal consistency, item-total correlations and correlated well with the CUCQ-12 questions. The stoma-specific questions correlated poorly with the generic QoL measures (EQ5D and VAS). EQ5D and VAS questionnaires do not address any stoma-specific QoL domains, such as body image. Therefore, this poor correlation results from the other generic QoL measures and the CUCQ-12 questions. We were able to identify 5 stoma-specific questions that best predict the QoL of patients with a stoma. The sample was not enough to carry out a full test-retest reliability analysis.

The CUCQ-32 is a derivative work of the Inflammatory Bowel Disease Questionnaire (IBDQ) [10,40,41], created and owned by McMaster University. Although there is a short version of the McMaster IBDQ (the SIBDQ) [40], the CUCQ-12+ is different in several aspects. The phrasing of the questions in the CUCQ-12+ has been customised for use in the UK. The response options of the CUCQ-12+ were simplified using a combination of close-ended and open 0–14 scores answers. The CUCQ-12+ also includes a question about urgency, which does not exist in the IBDQ-32 [41]. CUCQ-12+ includes supplementary stoma-specific questions. Therefore, the advantage of using the CUCQ-12+ is its simplicity and wide coverage of the symptoms of acute IBD.

Transition questions are considered advantageous to other methods in evaluating the QoL by directly addressing patients’ insights of change over time. They were used in several outcome measures studies [41,42,43,44,45]. We assessed responsiveness to change and minimally important change (MIC) using patients’ perceptions of their bowel condition via transition questions. Respondents with no changes in their bowel condition were included in the reproducibility analysis, while respondents with a change were included in the responsiveness and MIC analysis. Our test-retest validation was carried out over an average of a 3-m period. Future research is required to evaluate the reproducibility, responsiveness and MIC of CUCQ-12, using other disease indicators, such as endoscopic evaluation and clinical judgment, over a shorter period of 2–4 w as recommended in the literature [46]. 

The number of patients required to validate the QoL measures in the literature is lacking. The literature lacks guidance on the number of patients needed to confirm the QoL measures. However, a ratio of 5 or 10 patients per item was suggested [34]. Recent studies suggested that at least 100 patients are sufficient for a validation study [47]. Therefore our sample of 484 patients is more than sufficient to validate the 12 questions of CUCQ-12. However, the number of patients with a stoma was less than 100, which is suboptimal. Further validation is needed to confirm the validity of the supplementary stoma questions. 

To avoid any possible sampling bias, patients were recruited from 181 hospitals across the UK to ensure a good representation of patients and mirror routine clinical practice. Validation is an ongoing process, and the CUCQ-12+ needs to be validated in a varied demographic and clinical setting. In the meantime, the CUCQ-12 performed well in its present form and showed very good psychometric properties in the current study. 

The CUCQ-12+, a short QoL tool for patients with IBD, has demonstrated itself as a robust evaluation tool with positive internal reliability, reproducibility, validity, and responsiveness. Compared to current QoL measures, this new tool has proven acceptable to patients in the UK, concise and readily applicable in clinical practice. These qualities support the use of CUCQ-12+ in national IBD registries and databases and in audits, such as the UK IBD biological therapies audit [39].

## Figures and Tables

**Table 1:** The 32 items of the CUCQ-32 and stoma extension.

Rank	Questions Ranked by Percentage of Variance in the Total Score That They Explain	The Cumulative Percentage of Variance Explained
CUCQ-32 items		
	Unable to sleep well	45.9
	2. Felt tired	65.9
	3. Felt off food	74.0
	4. Had to rush to toilet	80.8
	5. Bloated abdomen	84.9
	6. Toilet immediately after emptying bowel	88.5
	7. Noticed blood in stools	90.4
	8. Felt generally unwell	91.7
	9. Bowels opened accidentally	93.0
	10. Toilet to empty bowel after going to bed	93.9
	11. Problem with wind	94.8
	12. Pain in abdomen	95.4
	13. Bowel condition affected leisure	96.2
	14. Irritable	96.8
	15. Full of energy (reverse coded)	97.4
	16. Runny bowel movement	97.8
	17. Felt sick	98.5
	18. Felt frustrated	98.8
	19. Opened bowels > 3 times a day	99.0
	20. Felt worried	99.2
	21. Avoid events with no toilet at hand	99.5
	22. Felt depressed	99.6
	23. Sex life affected	99.7
	24. Feeling happy (reverse coded)	99.8
	25. Felt embarrassed by bowel problem	99.8
	26. Prevented from going out socially	99.9
	27. Felt angry	99.9
	28. Felt the need to keep close to a toilet	99.9
	29. Felt upset	100.0
	30. Felt lack of sympathy from others	100.0
	31. Prevented from normal activities	100.0
	32. Felt relaxed (reverse coded)	100.0
Stoma supplementary items		
	Feeling less attractive	74.7
	2. Problems with stoma care	84.3
	3. Afraid others may hear about stoma	91.7
	4. Feeling less feminine or masculine	93.8
	5. Embarrassed	95.6
	6. Worried about leakage	97.2
	7. Worried of smell	98.2
	8. Dissatisfied with body	99.0
	9. Skin irritation	99.7
	10. Feeling less complete	100.0

**Table 2:** The characteristics of the patients in stage 2 who completed the baseline CUCQ-12 *.

Variable	Crohn’s Disease	Ulcerative Colitis	IBDU
Number of cases (n = 484)	382	76	26
Age			
<18	5	2	0
18–39	202	41	15
40–65	141	31	7
>65	34	2	4
Gender			
Male (%)	160 (42%)	41 (54%)	14 (54%)
Female (%)	222 (58%)	35 (46%)	12 (66%)
Non IBD co-morbidities	27	2	0
Smoking			
Current	82	5	2
Never	171	46	16
Ex-smoker	129	25	8
Generic QoL			
EQ5D utility score	0.65 (0.31)	0.69 (0.25)	0.63 (0.27)
EQ5D VAS	48.26 (28.55)	42.03 (30.24)	45.68 (27.56)
Had a postgraduate degree or qualification			
Yes	104	28	12
No	278	48	14
Disease severity			
HBI	5.19 (4.86)		
SCCAI		4.88 (4.02)	4.92 (3.97)

* Categorical data are presented as numbers (percentages). Continuous data are presented as means (standard deviation). EQ5D: EuroQol 5 dimensions (EQ5D) questionnaire. VAS: Visual Analogue Scale. HBI: Harvey–Bradshaw Index. SCCAI: Simple Clinical Colitis Activity Index.

**Table 3:** The clinical classifications of patients with IBD according to the Montreal classification (50).

	CD	UC	IBD-U
Colitis			
Proctitis		5	2
Left-sided (distal) colitis		41	14
Extensive (pancolitis)		30	10
Crohn’s disease 1. Location			
Ileal	96		
Colonic	117		
Ileocolonic	80		
No location was specified	89		
CD Perianal	67		
2. Behaviour			
Fistulating	73		
Inflammatory	255		
Stricturing	55		

CD: Crohn’s Disease, UC: Ulcerative Colitis, IBD-U: Inflammatory Bowel Disease Unclassified.

**Table 4:** Item total correlations for all the 12 items in CUCQ-12+.

Questions	Item–Total Correlations
On how many days over the last two weeks have you felt generally unwell?	0.79
2. On how many days over the last two weeks have you had to rush to the toilet?	0.76
3. On how many nights in the last two weeks have you had to get up to use the toilet because of your bowel condition after you have gone to bed?	0.67
4. On how many days over the last two weeks have you felt tired?	0.70
5. On how many days over the last two weeks have you wanted to go back to the toilet immediately after you thought you had emptied your bowels?	0.75
6. On how many days over the last two weeks have you felt pain in your abdomen?	0.74
7. On how many days over the last two weeks has your abdomen felt bloated?	0.67
8. On how many nights over the last two weeks have you been unable to sleep well (days if you are a shift worker)?	0.69
9. On how many days in the last two weeks have you noticed blood in your stools?	0.50
10. On how many days over the last two weeks have you felt off your food?	0.59
11. On how many days over the last two weeks, have you had a problem with large amounts of wind?	0.60
12. On how many days over the last two weeks have your bowels opened accidentally?	0.52
**Stoma supplementary questions**	
In the last two weeks have you felt less attractive as a result of your stoma?	0.80
2. On how many days over the last two weeks have you had problems with care for your stoma?	0.34
3. On how many days over the last two weeks have you been afraid that other people might hear about your stoma?	0.64
4. In the last two weeks, have you felt less feminine/masculine as a result of your stoma?	0.74
5. In the last two weeks, have you felt embarrassed because of your stoma?	0.80

**Table 5:** Pearson correlation coefficient between CUCQ-12 and with other measures.

Correlation of CUCQ-8 with	All Patients	Patients with CD	Patients with UC
HBI		0.45 *	
SCCAI			0.43 *
EQ5D	−0.48 *	−0.51 *	−0.50 *
VAS	−0.36 *	−0.37 *	−0.23 *

* *p* value < 0.005. Negative coefficients show that generic measures increase while disease-specific decrease. CD: Crohn’s Disease, UC: Ulcerative Colitis, EQ5D: EuroQol 5 dimensions (EQ5D) questionnaire, VAS: Visual Analogue Scale HBI: Harvey-Bradshaw Index SCCAI: Simple Clinical Colitis Activity Index.

## Data Availability

The data presented in this study are available on request from the corresponding author.

## Appendix A. Crohn’s and Ulcerative Colitis Questionnaire-12 (CUCQ-12) Plus

The following questions ask for your views about your bowel problem and how it has affected your life over the last two weeks. Please answer all the questions. If you are unsure about how to answer any question, just give the best answer you can. Do not spend too much time answering, as your first thoughts are likely to be the most accurate. If you do not wish to answer any of these questions, please leave it blank, and complete the details of the question and reason(s) why it was not answered.

1. On how many days in the last two weeks have you noticed blood in your stools?

…………………… days

2. On how many days over the last two weeks have you felt tired?

……………………. days

3. On how many days over the last two weeks have your bowels opened accidentally? …………………….. days

4. On how many days over the last two weeks have you felt generally unwell?

……………. days

5. On how many days over the last two weeks have you felt pain in your abdomen?

……….. days

6. On how many nights over the last two weeks have you been unable to sleep well (days if you are a shift worker)? ………………… nights (or days)

7. How many nights in the last two weeks have you had to get up to use the toilet because of your bowel condition after going to bed?

…………. nights

8. On how many days over the last two weeks have you had a problem with large amounts of wind? ………………….. days

9. How many days have you felt off your food over the last two weeks?

…………….. days

10. How many days have your abdomen felt bloated over the last two weeks?

………………. days

11. On how many days over the last two weeks have you wanted to go back to the toilet immediately after you thought you had emptied your bowels?

………………… days

12. How many days have you had to rush to the toilet over the last two weeks?

…………. days

If you did not complete any of these questions, please record the question number(s) below and, if possible, give a reason why it was not completed.

### Stoma Questions

Please answer the following questions only if you have a stoma. These questions ask you about your stoma and how it has affected your life over the last two weeks. Please choose only one answer for each of the questions. If you are unsure how to answer any question, give the best answer. Do not spend too much time answering, as your first thoughts are likely to be the most accurate.

1. In the last two weeks have you felt less attractive as a result of your stoma?

0. No, not at all.
1. Yes, some of the time.
2. Yes, most of the time.
3. Yes, all of the time.

2. On how many days over the last two weeks have you had problems with care for your stoma?..... days

3. On how many days over the last two weeks have you been afraid that other people might hear your stoma? ……… days

4. In the last two weeks, have you felt less feminine/masculine as a result of your stoma?

0. No, not at all.
1. Yes, some of the time.
2. Yes, most of the time.
3. Yes, all of the time.

5. Have you felt embarrassed in the last two weeks because of your stoma?

0. No, not at all.
1. Yes, some of the time.
2. Yes, most of the time.
3. Yes, all of the time.
